# Acute Calcific Tendinitis of the Longus Colli Muscle: Report of Two Cases and Review of the Literature

**DOI:** 10.7759/cureus.1597

**Published:** 2017-08-23

**Authors:** Ahmed Abdelbaki, Shady Abdelbaki, Neeraj Bhatt, Nishant Gupta, Shuo Li, Ahmadreza Ghasemiesfe, Yogesh Kumar

**Affiliations:** 1 Diagnostic Radiology, Bridgeport hospital/Yale Program; 2 Internal Medicine, Bridgeport hospital/Yale Program; 3 Radiology, Bridgeport hospital/Yale Program; 4 Radiology, Columbia University Medical Center; 5 Department of Radiology, Yale New Haven Health System

**Keywords:** acute calcific tendinitis, retropharyngeal effusion, dysphagia, neck pain

## Abstract

Acute calcific tendinitis (ACT) of the longus colli muscle is a rare cause of debilitating neck pain. The ACT is presumed to be an aseptic inflammatory process of the superior oblique tendons of the longus colli muscle. It is often confused with other more concerning conditions including trauma, epidural abscess, disc herniation, and neoplasm. We present two cases of ACT and a brief literature review to stress the risk of misdiagnosis. A 38-year-old male presented with neck pain and stiffness accompanied by dysphagia. Computed tomography (CT) scan was done and the diagnosis was secured by demonstrating calcifications at the C1-C2 level as well as the retropharyngeal effusion. A 53-year-old female was also complaining of neck pain and dysphagia. The CT scan demonstrated similar findings and the diagnosis was again clinched. Awareness of this unusual entity is essential to prevent unnecessary interventions.

## Introduction

Acute calcific tendinitis (ACT) is also otherwise known as retro­pharyngeal tendinitis or acute calcific prevertebral tendinitis. It is a self-limiting inflammatory condition presumably caused by calcium hydroxyapatite deposition in the longus colli tendon. The ACT was first described by Hartley, et al. in 1964 with calcium deposition just anterior to the cervical spine (C1-C2) [1]. The ACT often presents with acute posterior neck pain, dysphagia, odynophagia, stiffness and mild fever. Occasionally, a more sinister neoplastic or infectious diagnosis is proposed by ill informed radiologists or clinicians. Definitive radiologic diagnosis is often crucial to avoid unnecessary and often dangerous interventions. The diagnosis is usually established by the computed tomography (CT) scan of the neck. The CT is excellent in depicting prevertebral edema and calcific deposition associated with ACT [2]. We present two cases of ACT with calcium deposition and retropharyngeal edema, emphasizing on the salient imaging findings characteristic of this condition. Informed consent was obtained for this study.

## Case presentation

Case one 

**Figure 1:**
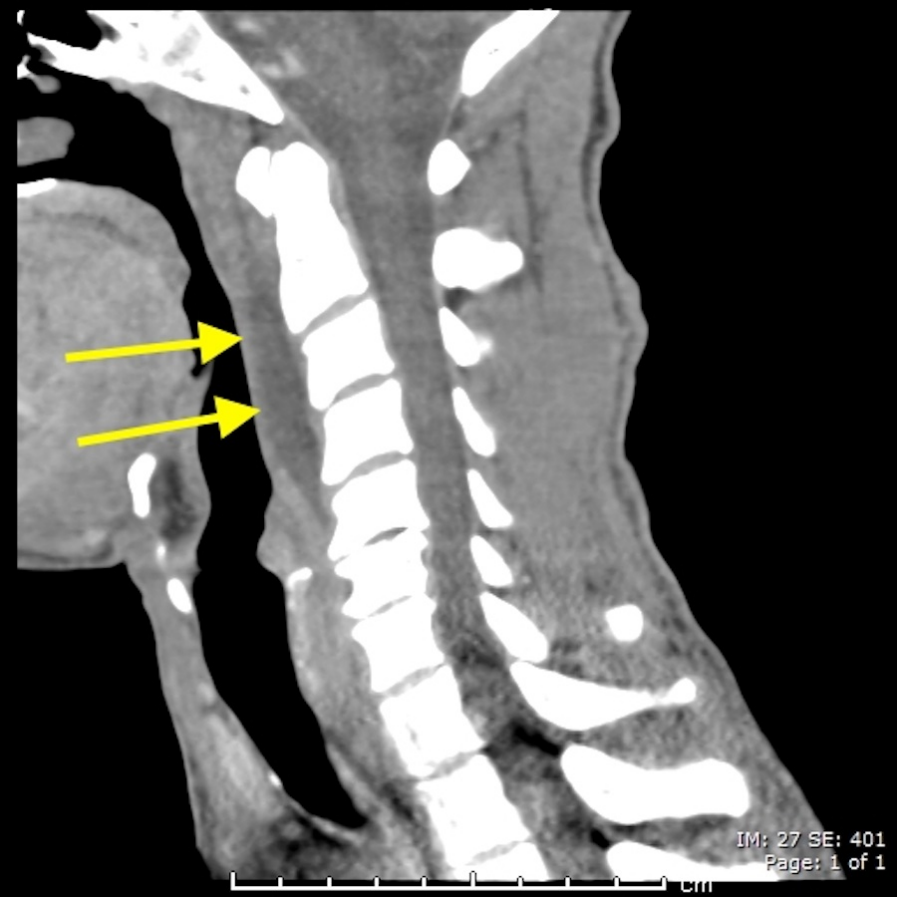
A 38-year-old male with acute calcific tendinitis. Sagittal contrast enhanced computed tomography (CT) scan of the neck (soft tissue window) demonstrating retropharyngeal effusion spanning from cervical spine C1 to C5 levels (yellow arrows).

**Figure 2:**
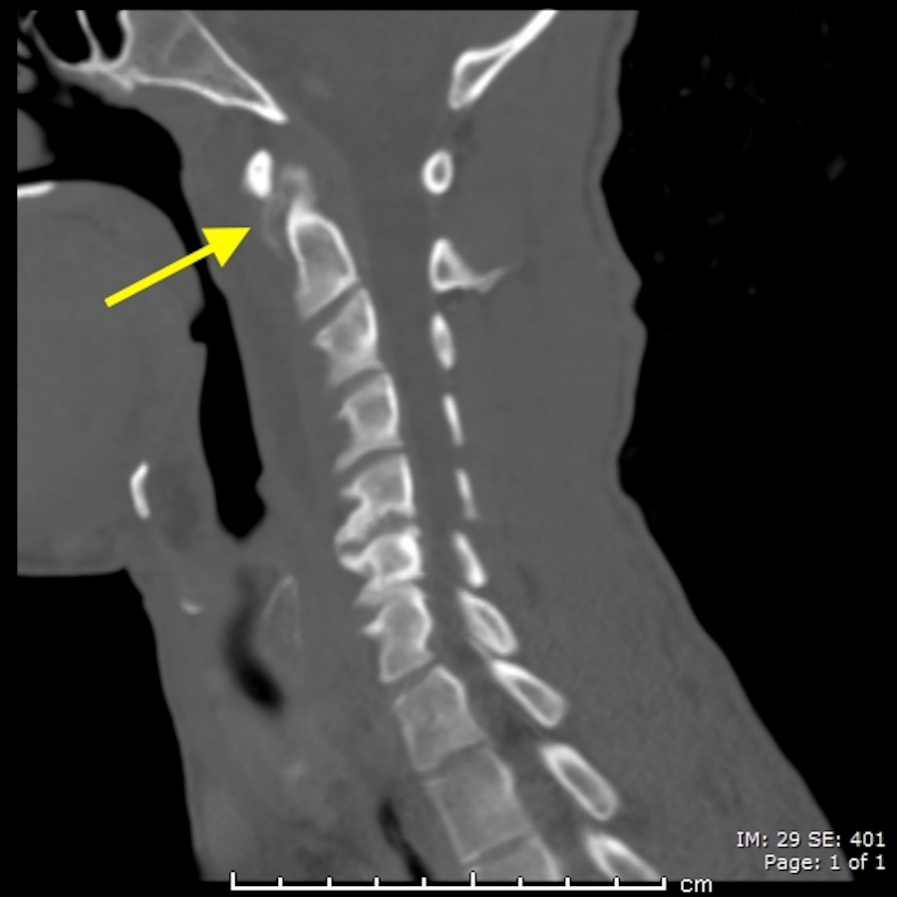
A 38-year-old male with acute calcific tendinitis. Sagittal contrast enhanced computed tomography (CT) scan of the neck (bone window) demonstrating amorphous dystrophic calcifications anterior to cervical spine C1 and C2 levels (yellow arrow).

**Figure 3:**
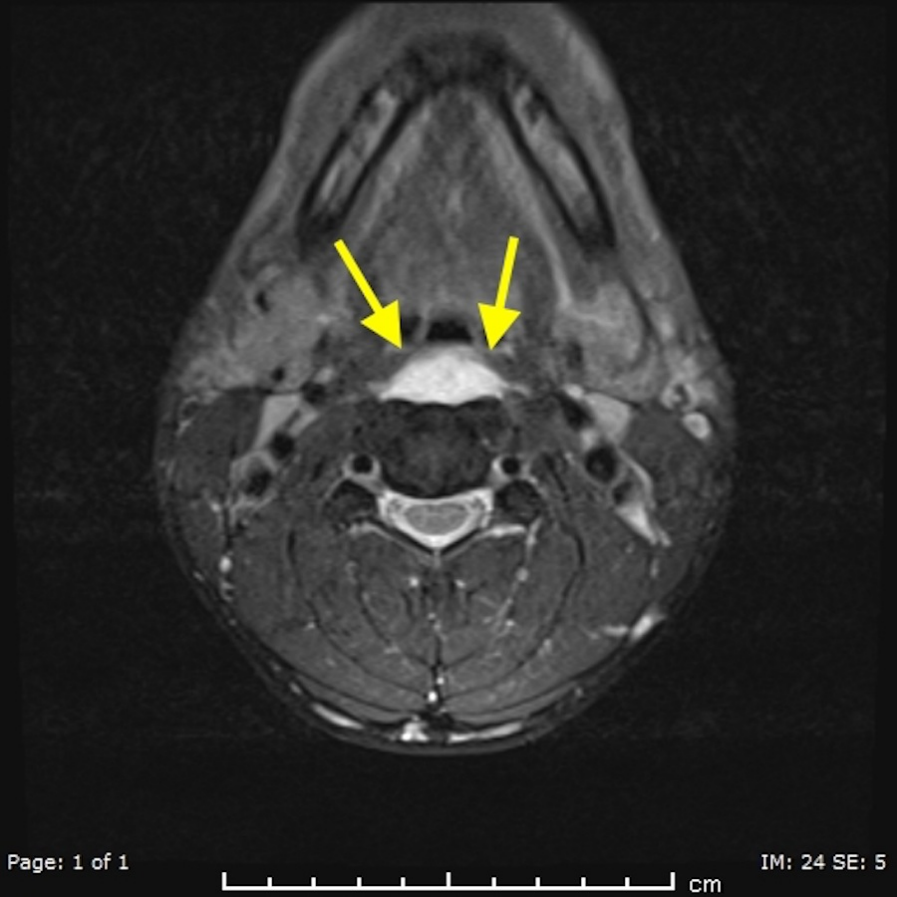
A 38-year-old male with acute calcific tendinitis. T2 weighted axial magnetic resonance imaging (MR) of the neck demonstrating retropharyngeal effusion (yellow arrows).

A 38-year-old male presenting with excruciating posterior left sided neck pain for four days, dysphagia and neck stiffness. The emergency department staff was initially concerned for abscess of the deep spaces of the neck. The CT scan of the neck with contrast was done and it revealed a prevertebral retropharyngeal low attenuation collection spanning from C1 to C5 (Figure 1). It also revealed dystrophic calcifications anterior to C1 and C2 (Figure 2). There was no enhancement of the fluid collection. Furthermore, there was no evidence of neck adenopathy or bone destruction to suggest a retropharyngeal abscess. Magnetic resonance imaging (MR) of the neck was done in an attempt to better characterize the disease process. It consolidated the CT findings (Figure 3) by demonstrating the retropharyngeal effusion. The patient’s symptoms improved significantly with few doses of Ketorolac and Acetaminophen after a brief period of hospitalization.

Case two

**Figure 4:**
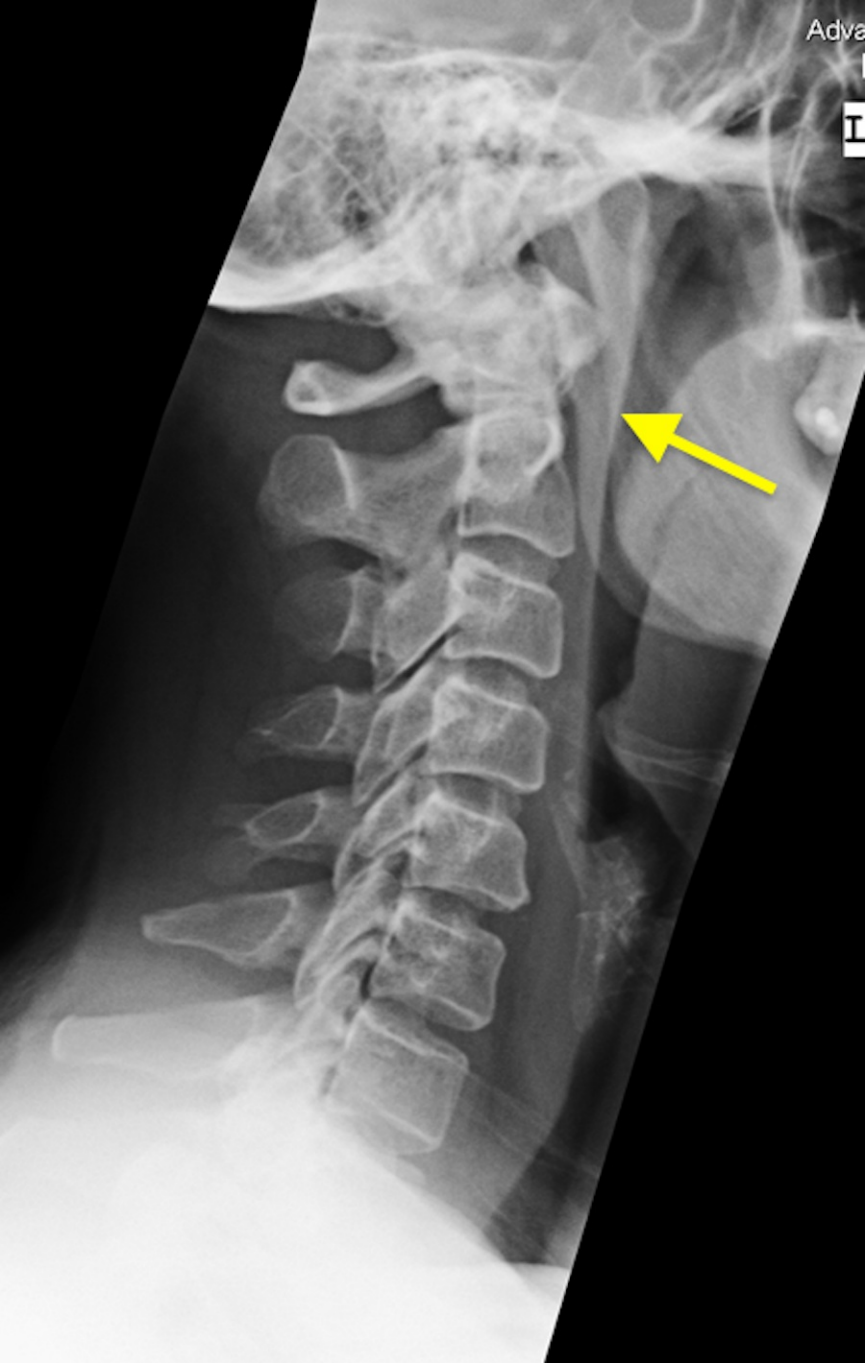
A 53-year-old female with acute calcific tendinitis. Normal lateral neck radiograph of the patient done in 2007. There is no evidence of pre vertebral soft tissue swelling or calcifications (yellow arrow).

**Figure 5:**
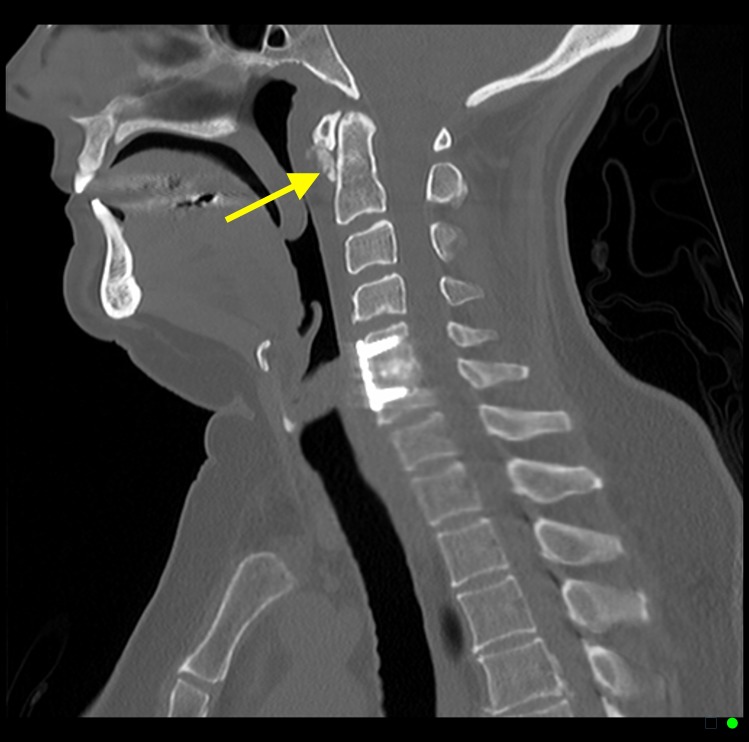
A 53-year-old female with acute calcific tendinitis. Sagittal non-contrast computed tomography (CT) scan of the neck (bone window) demonstrating amorphous dystrophic calcifications anterior to cervical spine C1 and C2 levels (yellow arrow).

**Figure 6:**
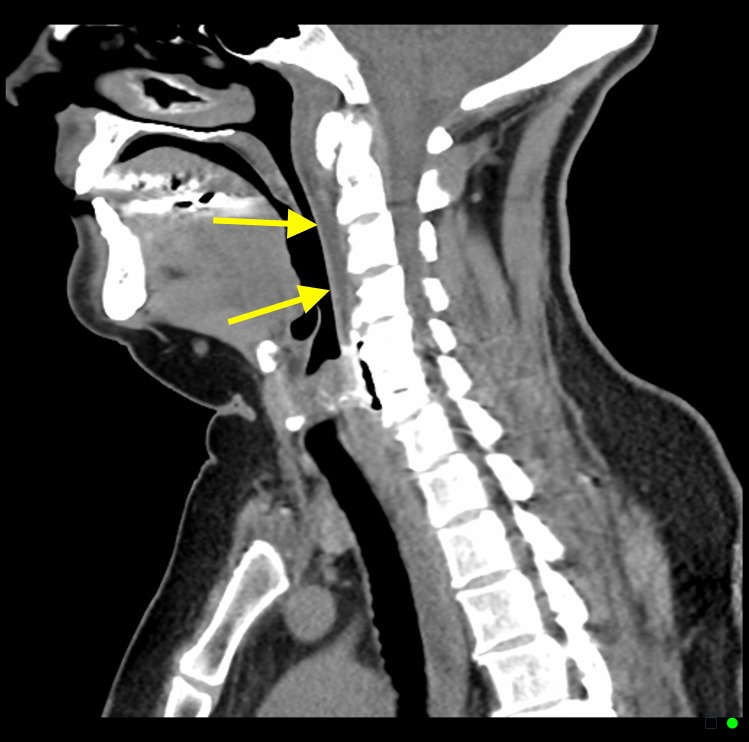
A 53-year-old female with acute calcific tendinitis. Sagittal non-contrast computed tomography (CT) scan of the neck (soft tissue window) demonstrating retropharyngeal effusion (yellow arrows).

A 53-year-old female presenting with neck pain and dysphagia for five days. The patient had normal lateral neck radiograph from 2007 (Figure 4). The CT scan of the neck without contrast was done and it demonstrated amorphous calcifications just inferior to the anterior arch of C1 (Figure 5). It also revealed retropharyngeal effusion (Figure 6). There was no evidence of neck adenopathy or bone destruction to suggest a retropharyngeal abscess. The patient symptoms improved drastically with nonsteroidal anti-inflammatory drugs (NSAIDs) and heat therapy.

## Discussion

The acute calcific tendinitis (ACT) was first described by Hartley, et al. [1] in 1964. It was also re-demonstrated by Ring, et al. [2] in 1994 as secondary to deposition of calcium hydroxyapatite in the longus colli muscle. The exact etiology of calcium hydroxyapatite crystal deposition is still poorly understood; however, some investigators hypothesize that repetitive ischemia, trauma, and degeneration contribute to the pathogenesis of ACT [3-4]. The longus colli muscle has three parts: upper-, vertical- and lower oblique fibers and is located in the prevertebral area. The ACT is an inflammatory process affecting the upper oblique fibers of the longus colli muscle. Upper oblique fibers originate from the transverse processes of C3–C5 vertebrae extending to the anterior tubercle of the atlas.

Our first patient was a 38-year-old male who presented with neck pain, dysphagia, and neck stiffness. The CT scan of the neck demonstrated the typical findings of non-enhancing retropharyngeal effusion and calcifications anterior to C1 and C2. The MRI of the neck was done as well and it demonstrated the non-enhancing retropharyngeal effusion. The patient’s symptoms resolved with NSAIDs, and he was discharged after a brief hospital admission. Our second patient was a 53-year-old female who presented with neck pain and dysphagia. The CT scan of the neck again demonstrated calcifications at C1 and C2 level and prevertebral effusion. The exact incidence of ACT is unknown, which is probably because the entity is commonly missed [5]. The disease is slightly more common in females and normally occurs in the third to sixth decades of life [2, 6]. The ACT typically presents with a triad of acute neck pain, neck stiffness, and odynophagia; however, the presentation is not always typical [2]. The ACT could be also associated with low-grade fever, elevated erythrocyte sedimentation rate and mild leukocytosis [2-3]. The CT scan is by far the imaging modality of choice for diagnosing this condition since the CT scan is excellent in depicting retropharyngeal effusion and calcific deposition at C1-C2 vertebral levels [2, 7]. Additionally, the CT scan has excellent sensitivity for distinguishing ACT from retropharyngeal abscess [3], by excluding features of retropharyngeal abscess like enhancing the fluid collection, inflammatory lymph nodes, and bone destruction. The MR imaging demonstrates the prevertebral muscle edema and corresponding fluid effusion; however, it has a poor sensitivity to detect calcific deposits [2, 8]. The CT scan is more sensitive than MRI for the diagnosis of ACT as it provides a better bony definition. The initial reports of this entity's pre-dated CT scan made effective use of plain radiography [1]. However, plain radiography may miss the calcific deposits and prevertebral effusion. Differentiating between ACT and other conditions with the similar clinical presentation, such as retropharyngeal abscess, meningitis, neoplasm, cervical disc herniation and fracture dislocation is critical. Retropharyngeal abscess is considered to be a notorious mimicker of ACT [2, 4]. Insufficient knowledge may result in misdiagnosis and unnecessary interventions such as antibiotic administration or incision and drainage of the retropharyngeal space. The ACT is a benign self-limiting entity [2-4]. The first line of treatment is non-steroidal anti-inflammatory drugs (NSAIDs). However, in severe cases, corticosteroids or opioids may be suggested [2-3]. It is also useful to establish immobilization with the soft cervical collar to avoid the aggravation of symptoms [2]. The vast majority of symptoms, usually resolve within few weeks from the initiation of treatment.

## Conclusions

The ACT is a rare benign cause of severe neck pain. The ACT is an underreported condition in the literature, clinicians, as well as radiologists, should be more aware of its existence. There are characteristic imaging findings of this entity. The CT scan of the neck is the imaging modality of choice. Our two patients had typical imaging findings of the ACT. Awareness of this unusual entity is essential to avoid unnecessary medical and surgical interventions.
